# The cycle of uncertainty: parents’ experiences of childhood epilepsy

**DOI:** 10.1111/1467-9566.12815

**Published:** 2018-10-23

**Authors:** Michelle Webster

**Affiliations:** ^1^ School of Law Royal Holloway University of London London UK

**Keywords:** Children, Epilepsy, Parenting/parents, Life course, Experience of illness

## Abstract

Uncertainty has been highlighted as an important aspect of experiences of chronic conditions generally and epilepsy in particular. However, there is little research exploring the extent to which uncertainty features in the experiences of family members or the form that this uncertainty may take. Drawing on in‐depth semi‐structured interviews with 27 parents who had a child with epilepsy, this article explores parents’ experiences of uncertainty and the way in which their views on childhood and epilepsy interacted and contributed to the uncertainties they experienced. It is argued that the occurrence of epilepsy during childhood shaped parents’ experiences as they used their ‘social clocks’ in order to interpret symptoms. Furthermore, parents described what has been termed a ‘cycle of uncertainty’. Indeed, the combination of epilepsy (a condition with various inherent forms of uncertainty) and childhood (a period in the life course that is seen as a time of development) meant that parents could not be sure which changes in their child were a result of the condition and which were a normal part of the ageing process. Overall, this article demonstrates that it is important to contextualise experiences of chronic conditions in relation to different stages in the life course.

## Introduction

The purpose of this article is to explore parents’ experiences of having a child with epilepsy and the way in which their perceptions of the condition and childhood interacted to shape their experiences. Epilepsy can manifest itself in a number of different ways and there is a high level of uncertainty inherent in the condition, particularly during childhood (Alarcón 2012). To date, there has been some research exploring individuals’ experiences of uncertainty when living with chronic conditions (Broom *et al*. 2015, Brown and de Graaf 2013, Honkasalo 2008, Kelly 1992, Lillrank 2003, Pinder 1988); however, less attention has been paid to the uncertainty experienced by other family members. Additionally, although there is some psychological research exploring the impact of having a child with epilepsy on parents’ mental health (Iseri *et al*. 2006, Lv *et al*. 2009), parental experiences have not been explored in detail from a sociological perspective. Consequently, this article explores whether and in what ways the occurrence of epilepsy during childhood can shape parents’ experiences of uncertainty. In order to do so, the article begins by reviewing the literature on the experience of uncertainty among those with chronic conditions, particularly epilepsy, and then moves on to the ways in which uncertainty can impact on parental experiences of caring for a child with a chronic condition.

## Uncertainty and the experience of chronic conditions

Uncertainty is an inherent aspect of medicine because when scientific advances resolve some uncertainties, they also create new ones (Fox 2000). Importantly, uncertainty in medical knowledge can have implications for the illness experience. Adamson (1997) acknowledges this and argues that ‘clinical’ and ‘existential’ uncertainty can influence one another. He defines existential uncertainty as a ‘form of uncertainty which is experienced privately by the individual patient upon the realisation that the future life of his or her mind, body and self is in jeopardy’ (1997: 134); and clinical uncertainty as the uncertainty of medical professionals who do not have the necessary knowledge to diagnose a condition or give a prognosis.

Existential uncertainty from the patient's perspective has been researched quite extensively and seems to be a common experience for those with a number of conditions. Indeed, experiences of uncertainty have been explored from the perspective of people with advanced‐stage cancer (Brown and de Graaf 2013), chronic pain (Honkasalo 2008), back pain (Broom *et al*. 2015, Lillrank 2003), colitis (Kelly 1992) and Parkinson's disease (Pinder 1988). For many of the individuals with these chronic conditions, it was uncertainty regarding the timing and severity of symptoms that was found to be most problematic.

However, diagnostic uncertainty, a particular form of clinical uncertainty, can also be stressful for individuals. This is likely to be because diagnoses are valued because they validate illness, offer an explanation and provide access to appropriate treatment (Jutel 2011, Jutel and Nettleton 2011). Consequently, when a diagnosis is not given this can be distressing (Jutel 2011). For instance, Dumit (2006) studied those with emergent illnesses and Lillrank (2003) explored the experiences of women with chronic back pain; both found that ongoing uncertainty as a result of not receiving a diagnosis was particularly troubling for these individuals.

Epilepsy is another condition that presents uncertainty. The diagnosis of epilepsy can be problematic as there are no definitive diagnostic tests and diagnosis is ‘complicated by the fact that many key symptoms and signs of epilepsy are intermittent and brief’ (Alarcón 2012: 6). Diagnosis in children is even more problematic as they may not be able to describe their seizures and because non‐epileptic paroxysmal events (seizures that are not caused by abnormal electrical discharges in the brain) are more common during infancy and childhood (Bagshaw *et al*. 2012).

Furthermore, similar to those with other chronic conditions there is a high level of uncertainty inherent in epilepsy related to the timing of symptoms. Seizures often occur without any warning (Schneider and Conrad 1983), which is the most troubling type of uncertainty for many individuals with epilepsy (Scambler 1989). Indeed, as Reis (2001) argues, for people with epilepsy the condition is not just about when seizures happen, times without seizures are also dictated by the condition, as individuals have to consider the possibility that a seizure could occur at any time. Additionally, there is the added uncertainty for children with epilepsy and their parents as to whether the child will grow out of the condition (Schneider and Conrad 1983).

Beyond the uncertainty experienced by individuals with a particular condition, it is also important to consider the way in which uncertainty impacts on family members. Most of the sociological research on the experience of epilepsy has focused on adults’ experiences, and where family members’ experiences have been explored the research is now quite dated (e.g. Scambler 1989, Schneider and Conrad 1983). Consequently, the literature on parenting children with a range of chronic conditions is drawn upon in the following section.

## Parenting children with chronic conditions

Given the multitude of ways in which an individual's daily life and can be shaped by chronic conditions, it is unsurprising that parents’ lives are also affected by having a child with a chronic condition. Indeed, Young *et al*. argue that ‘although they are not themselves ill, parents experience many of the consequences of chronic illness’ (2005: 97). Initially, diagnosis can be an important marker for parents that signals a turning point (Young *et al*. 2005). Indeed, Pitchforth *et al*., who studied families with a child with a nut allergy, described parents experiencing diagnosis as a form of ‘biographical disruption … in the life of the family’ (2011: 255). Diagnosis was experienced in this way because it was accompanied by a new set of responsibilities. In instances where a child's symptoms could be triggered by particular stimuli, such as when a child had asthma or an allergy, parents described being constantly vigilant about the child's activities and their environment in order to avoid triggers (Nocon and Booth 1990, Pitchforth *et al*. 2011). The uncertainty associated with the timing of symptoms sometimes resulted in considerable amounts of worry for parents with a child with asthma, either due to the potential severity of symptoms or the fact that they may not be there to care for their child (Barton *et al*. 2005).

Parents’ routines may also change as a result of the uncertainty associated with the timing of symptoms; for instance, parents sometimes had to cancel social engagements or take time off work and they gave additional consideration to holiday destinations or ruled some out altogether (Barlow and Ellard 2006, Barton *et al*. 2005, Hill and Zimmerman 1995, Nocon and Booth 1990, Timmermans and Freidin 2007). Furthermore, when symptoms could occur at night, parents often experienced sleepless nights and felt fatigued the following day (Barton *et al*. 2005, Nocon and Booth 1990) or they adjusted their sleeping arrangements so that they could sleep in the same room or bed as the child (Barton *et al*. 2005, Williams *et al*. 2000).

Although this body of literature highlights the impact of uncertainty on parents, the way in which parents experience uncertainty is yet to be fully conceptualised. Building on Schneider and Conrad's (1983) and Scambler's (1989) research that highlights the implications of uncertainty associated with the timing of seizures among adults with epilepsy, the purpose of this article is to explore parents’ experiences of uncertainty resulting from having a child with epilepsy. The methodological approach taken in order to explore this is outlined in the section below.

## Methodology

The data presented within this article are drawn from a broader study focusing on the experience and management of childhood epilepsy within the family. A qualitative approach was employed to explore, in detail, parents’ experiences of having a child with epilepsy. The research was advertised through seven UK‐based charities that placed adverts on their websites, online forums, social media pages and in their newsletters. All those who volunteered to take part in the study were included in the sample and the research was re‐advertised and data were collected until saturation point was reached.

In total, 24 families took part in the research and one or both parents in 23 of the families participated in an in‐depth semi‐structured interview. The data presented below comprise the views of 23 mothers and four fathers. These parents had a child with epilepsy aged 3–13 years; 14 of the children were male and eight were female. These families were living in households that ranged in size from three to six people. The majority were two‐parent families, two of which were stepfamilies. One family was a single‐parent family and another had for a long time been a single‐parent family but had moved in with the mother's long‐term partner a few months before the interview. Of those who gave information about their income, the majority had an income above the national median (Pike 2011), with only two families stating an income below the national median.

Nineteen of the 23 families where the parent(s) took part in a semi‐structured interview were living in mainland UK; the majority of these parents self‐identified as White British, one identified as White European, one was Irish, one was from Continental Europe, one identified specifically as being Scottish and one parent was Asian (foreign‐born). The broader study explored the use of two different treatment methods and due to difficulty recruiting families using one of these treatments, parents from outside of mainland UK were included in the sample. Consequently, four families were from non‐mainland UK, Eastern Europe and Western Europe.

All parents from mainland UK were interviewed face‐to‐face and the other four interviews were conducted over the phone or via Skype and one was an email interview. Additionally, the majority of the face‐to‐face interviews were conducted with participants in their own homes, with the exception of one, which was carried out in a café at the participant's request. The presence of both parents in four of the interviews undoubtedly shaped the data as there were sometimes disagreements (Valentine 1999), but this did provide an insight into the perspectives of both parents as well as shared experiences (Morris 2001). Although the experience of mothers and fathers can differ, the findings presented in this article relate to the experiences of all participants. Indeed, although only four fathers participated in the research, those that did took an active role in the interviews and articulated experiences that aligned with those of mothers.

The parents’ interviews lasted between half an hour and two hours, with 14 of the 23 interviews being roughly one hour in length. Furthermore, parents gave very detailed answers and often used stories to illustrate their points. The interviews were guided using an interview schedule, which included the following topics: the history of their child's epilepsy (i.e., type of seizures, first seizure, diagnosis and the different treatments used), day‐to‐day routines associated with the condition and how parents felt having a child with epilepsy had impacted on their family life.

All interviews were audio‐recorded and transcribed *verbatim*, with the exception of the email interview. The data were then coded using NVivo and analysed using a constructivist grounded theory approach (Charmaz 2006). In contrast to Glaser and Strauss’ (1999) grounded theory, a literature review was conducted prior to carrying out the interviews in order to gain an understanding of previous research on similar topics. But, in accordance with Glaser and Strauss (1999), themes were developed using the constant comparative method throughout the data collection phase, emerging themes were drawn upon in later interviews to fill gaps in the analysis, and participants were recruited until categories became saturated.

Ethical approval was granted by the Centre for Criminology and Sociology's departmental ethics committee at Royal Holloway, University of London prior to beginning data collection. All participants gave informed consent and participants and their family members are referred to using pseudonyms.

## Findings

The findings presented here primarily focus on the uncertainties described by parents that related to epilepsy during childhood. This section begins by exploring the problems parents faced in relation to recognising symptoms. Following on from this, the focus shifts to diagnosis and the uncertainty parents described in relation to this process. Next, the ‘cycle of uncertainty’ will be discussed; parents explained how they were unsure about which changes in their child could be attributed to the child's age and which were a result of seizures, medications or the condition more generally. Lastly, the impact of epilepsy on the way in which parents imagined their children's futures will be discussed.

### Recognising symptoms

Parents in 13 of the 15 families where onset of the condition was spoken about in detail described how they initially did not recognise the child's seizures as being ‘a seizure’ or cause for medical concern. Many of these parents explained that they had initially interpreted the child's symptoms by drawing on an understanding of childhood behaviour and concluded seizures were ‘just one of those things children do’. For instance, many parents expressed the opinion that children sometimes do ‘strange’ things, daydream or play games, and this is what seizures tended to be interpreted as. Bury and Holme (1991) introduced the concept of the ‘social clock’, arguing that people have an idea of when in the life course certain conditions will occur. Based on parents’ comments, the concept of the social clock can also be applied to explain how people originally interpret symptoms, therefore supporting Jutel's argument that ‘a collective cultural position determines which symptoms we see, [and] which we brush off as insignificant’ (2011: 61). Here, parents drew on assumptions surrounding childhood behaviour in order to explain children's seizures, rather than positioning this behaviour in the medical domain.

Parents did not always quickly arrive at the conclusion that their child's symptoms were potentially a medical problem. Indeed, parents often began to pay closer attention when a behaviour continued or increased in frequency, leading to uncertainty regarding whether this behaviour was in fact ‘normal’. For example, Emma spoke about not originally recognising her son's behaviour as a seizure.

Emma He started what is absences, but we didn't really know at the time. Just stopping, eye rolling a lot.

I OK. And so you took him to the GP?

Emma Yeah, not for a while though. I wanted to keep an eye on it and see if it wasn't just one of those things that kids do.

Emma's extract demonstrates that she was unsure about what her son was doing and why. This period of uncertainty is concurrent with the first stage of biographical disruption where bodily states are given more attention than usual; alternatively described by Bury as the ‘“what is going on here” stage’ (1982: 169). However, in contrast to Bury's (1982) work, here the onus was on parents, rather than the individual with the condition, to recognise symptoms and seek medical advice, illustrating that family members who are not presenting symptoms can also experience this phase of uncertainty. Additionally, even when parents had placed their child's behaviour in the medical sphere they could still not be sure what was happening. The uncertainty inherent in this experience was best summarised by Heather when she said ‘we didn't know at first what was happening at all or what it was or why it was’.

When parents came to view children's behaviours as seizures, the uncertainty relating to their child's behaviour was resolved. However, gaining a diagnosis and resolving this uncertainty was not always simple due to diagnostic uncertainty, as we see below.

### Diagnostic uncertainty

Given the diagnostic uncertainty that is evident in the clinical literature, it is not surprising that prolonged uncertainty surrounding diagnosis featured in 11 of the 23 families’ stories. Even in the other 12 families where parents reported that a diagnosis was reached relatively quickly, there was often still a short period of uncertainty.

Seven of the 23 children were said to have been misdiagnosed and two of the children were reportedly misdiagnosed more than once. Parents recalled the children being incorrectly diagnosed as having an eye condition, vitamin B6 deficiency, another vitamin deficiency, a nervous tick and three were said to have been diagnosed with night terrors. Although not specific diagnoses, parents claimed that consultants also described children as ‘a late developer’ and ‘attention seeking’ when parents sought medical advice. It is important to acknowledge that the data represent parents’ narrative constructions of their children's diagnoses and their interactions with mediation professionals, rather than fact. However, if these parents’ recollections are true, it suggests that, similar to the parents, consultants also evaluated children's symptoms using their social clocks. For instance, night terrors are only experienced by children, which may explain why this diagnosis was given. Additionally, children being seen as ‘a late developer’ and as ‘attention seeking’ are likely to be linked to the child's stage in the life course.

Further complicating the process of receiving the diagnosis was the nature of the condition. Seizures are intermittent and people are often unconscious during them; as a result, if they are not witnessed by another person it can be hard to determine what is wrong with someone based purely on their post‐ictal phase (the period following a seizure). This is likely to be a problem for the diagnosis of adult as well as childhood epilepsy. However, the fact that this condition had developed during childhood did add to the diagnostic uncertainty. For one family this became more apparent as the child got older. Chelsea, Robert and Marie's daughter, had started having seizures when she was around 4 years old; she was medicated and became seizure free and was then weaned off her medication at the age of seven. However, after being discharged from the hospital her seizures started again. Below Robert is describing the difference between the two periods of seizure occurrence.

Well, this is the difference, when it first happened she was four, five years old so she couldn't articulate what was going on. She couldn't explain what was happening. So you could just see the outside.

Chelsea's parents speculated that they had not noticed her seizures until they had become more pronounced because she had not been able to describe her seizures when they first occurred. Supporting this contention, when Chelsea started having seizures again she said that her arm kept going numb; when Marie and Robert reviewed videos of Chelsea having seizures when she was four years old they could see that she would use her left arm to pick up and move her right arm and thought this was probably because her arm had been going numb but she had not been able to tell them. This example shows that the occurrence of the condition during childhood meant that symptoms were sometimes harder for parents to interpret and for consultants to diagnose because younger children either did not have the vocabulary to describe their seizures, or possibly did not think these sensations were unusual.

For some parents the diagnosis of epilepsy was a shock. However, similar to arguments made previously regarding adults’ experiences of diagnosis (Jutel 2011, Jutel and Nettleton 2011, Lillrank 2003), for other parents it was a relief to finally have a label for their child's condition and to know that they would then be treated. Parents who fell into the latter category were often those who described experiencing a long period of uncertainty waiting for a formal diagnosis. Critically, however parents felt about their child's diagnosis they tended to see this as an end point to some of their uncertainty – they could now label the child's seizures as a symptom of epilepsy, and often as a particular type of seizure.

However, diagnosis did not end all uncertainties experienced by parents. Indeed, diagnosis often created new uncertainties, such as causal uncertainty (discussed in Webster 2016), and there were also ongoing symptomatic uncertainties, which are outlined below.

### Symptomatic uncertainties

Parents described three types of symptomatic uncertainty; they lived with ongoing uncertainty regarding the timing of seizures, the severity of their child's next seizure and also, on occasion, whether the child had had a seizure.

Regardless of whether children's symptoms were well controlled or if they were still regularly having seizures, parents explained that they were uncertain regarding when their child's next seizure would occur. For instance, Catherine explained:

They [seizures] happen all through the day and to a greater or lesser extent just depending on, I don't know. I don't know what. You can't predict epilepsy, can you? That's the hardest part about it.

Catherine's uncertainty regarding the timing of her daughter's seizures is clearly illustrated in this extract; she begins to talk about what causes fluctuations in the frequency of her daughter's seizures but realises that she has no way of explaining this. Moreover, the majority of parents in this study agreed with Catherine that the unpredictability of the condition was a particularly hard aspect to deal with. The prevalence of discussions relating to this type of uncertainty is probably why it is the main type of uncertainty mentioned in previous studies on people with epilepsy (Reis 2001, Scambler 1989, Schneider and Conrad 1983).

It was not just children who had seizures every day whose symptoms were seen as unpredictable. For instance, parents of children who were currently seizure free could still not be sure how long seizure control would last. As Zara explained:

He's not having any fits at the moment … but we are kind of waiting that when he puts weight on, and also when he hits puberty, you know, we don't know what's going to happen then.

Here, Zara explains that she is ‘waiting’ for her son's symptoms to reoccur, but she cannot predict exactly when this will happen.

Furthermore, parents discussed being uncertain regarding how severe their child's next seizure would be and, consequently, parents could not be sure how long the child's post‐ictal phase would last. For example, when Ruth was asked whether her daughter experienced a post‐ictal phase she said that she might be ‘a bit spaced out the next day’, but if her daughter had required emergency medication then it would take her much longer to recover. For instance, Ruth recalled one instance when her daughter ‘slept for 18 hours afterwards’. Therefore, although every child's epilepsy and seizures differed from one another, all were seen by parents to be inherently unpredictable.

The third type of symptomatic uncertainty that many parents experienced was that they were sometimes unsure about whether their child had had a seizure. Many of the children did not know when their seizures were going to occur; similarly, the children were, at times, not sure whether they had had a seizure. Some children had learnt to interpret feelings following a seizure as an indication that they had probably had a seizure, however, not all children did this. It seemed that younger children found making this link the hardest, possibly because they had the least time to become familiar with their symptoms or because, according to parents, younger children had the least understanding of their condition. For example, Emma explained that her son:

Didn't used to say he'd got any warning but now and again, quite recently he'll say ‘I feel a bit dizzy’. So we'll just be careful. I presume that is, you know, that he feels like he's having a few seizures or something. But when I ask him he'll say ‘no, I don't know’. So maybe he's just learning himself.

Children's uncertainty about whether they had had seizures resulted in parents often feeling uncertain as well; if parents suspected their child had had a seizure, the child was not always able to give them a definitive answer.

Related to uncertainty about whether children had experienced seizures was parents’ uncertainty regarding what was associated with the condition. This particular type of uncertainty is referred to here as the ‘cycle of uncertainty’, and is discussed in the following subsection.

### The cycle of uncertainty

Of the 23 children, 20 had experienced changes in their symptoms and those who experienced multiple types of seizures had often started with one type and others had developed later on. For instance, Donna explained that her son had originally experienced absences^1^ , but later developed tonic–clonic seizures^2^ and more recently had started jerking movements, which were becoming more ‘noticeable’. Even parents whose children had not developed new types of seizures often found that the way in which seizures presented themselves had changed. For example, when Steve was speaking about reading back through the seizure diary he and his wife kept, he said:

The earlier ones you can read and understand that it's a simple, you know, he just stops and stares and starts looking up to the left; whereas now they're [seizures] doing so many different things.

Consequently, children's epilepsy was not seen to be stable and these changing symptoms contributed to parents being on, what will be termed here, a ‘cycle of uncertainty’. This cycle of uncertainty appeared to be related specifically to *childhood* epilepsy because neither the condition nor childhood were deemed to be stable. Each time the child presented a new behaviour, parents began on the cycle – firstly, parents tried to determine if the behaviour was normal for the child's age or whether it was related to the condition; secondly, if they thought it did relate to the child's epilepsy, they wanted to know specifically how to define it. Often the only way for parents to feel this cycle had ended was for the behaviour to be diagnosed by a consultant as a side effect of treatment, a result of the condition or as a certain type of seizure.

The first stage of this cycle of uncertainty was illustrated by many parents who spoke about currently being uncertain about whether some of their child's behaviour was normal for their age or related to the condition or its treatment. Sarah gave one example of this when she was discussing her son's performance at school.

So honestly, I don't know whether it's him, whether it's a side effect of the epilepsy or what, but definitely his [attainment] levels were lower at the beginning of the year, kind of got them back up again and they've gone back down again. And I'm unsure as to why.

This extract shows that the first stage on the cycle of uncertainty was very similar to the uncertainty parents had initially described when they were talking about the onset of the child's condition. However, rather than trying to determine purely whether a behaviour was a cause for medical concern, parents like Sarah were unsure about whether what they had noticed was linked in some way to the child's existing diagnosis. Consequently, the child's diagnosis of epilepsy and their ongoing treatment for the condition complicated parents’ explanatory tools. In the past, childhood had been their main frame of reference, but now epilepsy was an alternative.

During Steve and Nicola's interview, they questioned whether their son's ‘stroppy’ moods were linked to his medication or were a sign that a seizure was imminent; however, they also commented that they had not had a teenager before so they were not sure what was ‘normal’ for his age. This deliberation suggests that they were also experiencing the first stage on the cycle of uncertainty. Additionally, Steve suggested that this was a problem that ‘someone needs to come up with an answer for’. It seems that, again, much like parents’ initial uncertainties regarding what children's seizures were, parents were relying on medical professionals to answer their questions as a way of ending their uncertainty.

The second stage on the cycle of uncertainty was reached when parents decided for themselves that their child's behaviour was a result of their condition without confirmation from medical professionals; this did not mean, however, that their uncertainty was entirely resolved. For instance, Samantha explained that she carried a small diary with her where she would note anything ‘unusual’ that happened to her son. Below, she is describing one regular occurrence that she was monitoring.

Quite often he'll just come really pale and he'll say ‘I don't feel good. I feel sick’. And then he'll say ‘I feel odd. I feel really odd’. And then after about 10 minutes or half an hour, and he has a lie down on the sofa, he's fine and back to being hyperactive and playing or whatever. So I don't know whether they're auras or that's a type of partial seizure. And again, when you talk to the doctors they don't really say anything. They just go ‘OK’ and write it down. And that's it.

Here, Samantha describes how she had tried to explain these occurrences by drawing on her son's original diagnosis. Furthermore, she had researched different types of seizures. However, Samantha was still uncertain as to what type of seizure this might be. What we see in this section is that parents often drew on their interactions with medical professionals in order to illustrate and emphasise their experiences of ongoing uncertainty. It is clear that Samantha was hoping a medical professional would be able to end her uncertainty, but she did not deem the answers she had been given to be satisfactory. Indeed, parents felt that, by themselves, they could only come close to the end of the cycle – they could never be completely certain without confirmation from a medical professional. For instance, Samantha also commented:

I don't know … sometimes we put things down to epilepsy that perhaps aren't. And sometimes we don't put things down to it that perhaps are. But nobody's got any definitive answers at all. That's the most frustrating part really.

Consequently, despite parents’ suspicions the only definitive end to this cycle of uncertainty was for the behaviour to be diagnosed by a consultant as a side effect of treatment, a result of the condition or a certain type of seizure. As this reveals, as Jutel (2011) has argued, despite the lay public being better informed, the medical profession still maintains its authority as the process of diagnosis gives medical professionals the ‘last word’.

However, this is not to say that parents uncritically accepted medical professionals’ opinions. There were many instances when parents described raising concerns with their child's consultant and being told that there was no link to the condition, but parents were not always convinced. However, they could still not be entirely certain without having their suspicions, albeit strong suspicions, confirmed by a medical professional. For instance, Heather said she was not sure whether her son's medication was resulting in side effects or if his behaviour was related to his age. She recalled raising this concern with an epilepsy nurse, who had asked the consultant and she was told:

Through the epilepsy nurse, she came back to me and said ‘oh, she [the consultant] says it's nothing to do with the medication because he's been stable for a while’. That's something I don't agree with. I think it can affect him off and on. As I said, if there's anything else going on it could trigger something. Or maybe if he has a cold and on the medication that might make it worse. I don't think you can say categorically ‘that medication has nothing to do with him’.

This extract illustrates that, as Jutel (2011) and Jutel and Nettleton (2011) have argued, lay people are now in a position to contest diagnoses or the opinion of medical professionals. However, it also reveals that Heather's cycle of uncertainty could only have come to a close if she felt her son's consultant had agreed with her explanation that Ross’ medication was contributing to his behaviour. Without this confirmation, although convinced her son's behaviour was linked to the condition, Heather was not completely certain regarding the way in which it was associated. Consequently, parents often became stuck at various points on this cycle of uncertainty because they felt they had unanswered questions and, consequently, could not feel fully informed about their child's condition. Furthermore, this cycle of uncertainty could potentially begin again in relation to a new symptom or behaviour at some point in the future.

In this subsection it has been illustrated that because neither childhood nor epilepsy were seen to be stable, parents often became stuck in a cycle of uncertainty where they could not be sure which of their child's behaviours were related to the condition. However, it was not only the present, but also the future that was seen to be uncertain. The focus of the next subsection is parents’ discussions of uncertain futures.

### Uncertain futures

Although most people feel that the future is somewhat uncertain, parents of children with epilepsy talked about how they felt the condition contributed to the future feeling even more uncertain. Parents sometimes questioned whether their child would have the condition for the rest of their lives, but they more frequently talked about the impact of the condition on the child's future and the possibility of a limited future.

When epilepsy occurs during childhood there is sometimes the possibility that children will grow out of the condition (Schneider and Conrad 1983). Parents in 11 of the 23 families reported that they had been told that this would not, or was unlikely to, happen. However, the other 12 families could not be sure whether epilepsy would feature in the child's future. For example, Marie said:

We can't control it, we can't change it. We don't know how long it's going to last for. We don't know whether she'll ever grow out of it or whether she'll have it for the rest of her life.

For Marie, and other parents in a similar position, the future was uncertain because they could not predict how long their child's condition would last. As Carol said, ‘maybe it will stop as quick as it started’.

Not only did parents not know how long the condition would last, they also did not know how it would change over time. For example, Anita explained that the type of epilepsy her daughter had had changed since she was diagnosed. She also said ‘I'm very conscious that now it's changed to generalised epilepsy^3^ , you know, it might change, she might develop a different type of epilepsy’. Therefore, even when parents could be sure that epilepsy would feature in their child's future, they could not be sure what form this would take.

An additional uncertainty relating to childhood epilepsy was what would happen when the child reached puberty. Puberty and teenage years featured in many parents’ discussions and always seemed to be perceived as a problematic time during a person's life, which, for their children, would be complicated further by epilepsy. Similar to Zara's comment above, Marie noted:

From what I've read, puberty and the hormones and everything like that can cause havoc … It can make them [seizures] more frequent or it can make them a bit more unexpected.

Consequently, parents were often nervous about their children reaching puberty because they could not be sure how this phase in their child's life would be affected by epilepsy and vice versa.

Not only did parents consider how the condition might develop, they also considered what this meant for their child's future life choices. For instance, Steve wondered how his son would manage if he chose to go to university and live away from home. Three parents also questioned what impact the condition would have on their child's future job prospects, and four talked about how their child may never be able to drive.

All of the extracts above relate to parents considering a future for their child and the extent to which epilepsy would feature in that future. However, parents in 13 of the 23 families also questioned how much of a future would exist, and whether that imagined future might be cut short. For instance, parents spoke about the fact that epilepsy could, in some instances, lead to death, as can be seen in Kelly's extract below.

You constantly worry about him. Especially with this syndrome because you know that any seizure could be, you know, could either cause irreparable brain damage or he could have a seizure that kills him, or he could suffer from SUDEP [Sudden Unexpected Death in Epilepsy] … They're all worries.

Consequently, the occurrence of this condition during childhood sometimes conflicted with notions of childhood as a time of development, as epilepsy posed an existential threat to the child.

## Discussion

This article has demonstrated that uncertainty was a significant aspect of parents’ experiences of having a child with epilepsy. In accordance with Adamson (1997), it has been shown that it is helpful to break down the concept of uncertainty to reveal a deeper understanding of people's experiences of uncertainty. However, here experiential uncertainty has been broken down further to reveal the multitude of uncertainties experienced by parents of children with epilepsy. As has been noted in previous studies on epilepsy, one of the most common forms of uncertainty associated with the condition related to the timing of seizures (Reis 2001, Scambler 1989, Schneider and Conrad 1983). However, additional types of uncertainty that were prevalent here related to the onset of the condition and having to initially interpret symptoms and diagnostic uncertainty (previously mentioned by Schneider and Conrad 1983). Furthermore, two additional types of symptomatic uncertainty (relating to the severity of the next seizure and whether a seizure had occurred) were frequently experienced.

Importantly, a number of these types of uncertainty were not only shaped by the uncertain nature of the condition, but also by the occurrence of the condition during childhood. Firstly, many parents explained that they had initially felt uncertain about whether the child's seizures were normal childhood behaviours or a cause for medical concern. Indeed, it was evident in parents’ descriptions that children were viewed as ‘becomings’ (Hockey and James 1993); they were seen to be progressing through a period of change. Parents also clearly viewed children as distinct from adults (Hockey and James 1993), as it was often noted that children sometimes do ‘strange’ things. Consequently, this perception of childhood clearly impacted on the way in which parents initially interpreted children's symptoms. It has been shown that Bury and Holme's (1991) concept of the ‘social clock’ could be utilised and extended to explain this experience; it can be seen that the child's stage in the life course was taken into consideration when interpreting their behaviour. Secondly, the occurrence of the condition during childhood also contributed to diagnostic uncertainty, particularly among younger children, as they were not always able to recognise or describe their symptoms; in this sense they were seen as distinct from adults or as not fully complete (Hockey and James 1993).

Furthermore, the cyclic experience of uncertainty described by parents resulted from the intersection between epilepsy (a condition with various inherent forms of uncertainty) and childhood (a period in the life course that is seen as a time of development). As neither the condition nor childhood were seen as stable, parents could not be sure which changes in their child were associated with the condition and which were normal for their age, mirroring the uncertainty experienced around onset. If parents did believe the child's behaviour was related to the condition they could not define specifically what type of seizure this might be without receiving a diagnosis from a medical professional. As a result, parents often became stuck on this cycle of uncertainty. Furthermore, it seemed that this process would start again every time the child presented a new behaviour or symptom. This cycle of uncertainty is likely to be specific to family members’ experience of *childhood* epilepsy, rather than epilepsy as a condition more broadly, because of current conceptualisations regarding childhood being a time for development (Hockey and James 1993). However, the cycle of uncertainty may also be experienced by parents who have children with other chronic conditions that change over time.

Lastly, when parents considered their child's future in accordance with their condition, this raised additional uncertainties. Indeed, puberty was seen as a time in the life course that is particularly turbulent and parents were sometimes anxious about the interaction between epilepsy and this developmental stage. Furthermore, some parents also spoke about the possibility of a limited future for their child, which directly contrasted with the notion that children are ‘becomings’ (Hockey and James 1993) and are developing into their future adult selves.

This article has also contributed to the literature surrounding experiences of living with epilepsy. Research to date has tended to focus on the experience of those with the condition (Scambler 1989, Schneider and Conrad 1983). However, this study has illustrated that other family members, in this case parents, also experience uncertainty. Additionally, by focusing on childhood epilepsy, the interactions between the condition and this particular stage in the life course has uncovered uncertainties that appear to be specific to the occurrence of this condition during childhood, for example, the cycle of uncertainty.

Overall, through the analysis presented, this article has highlighted that experiences of chronic conditions, in this case epilepsy, need to be contextualised in relation to different stages in the life course. Indeed, parents’ experiences were clearly shaped not only by the condition, but also by current societal notions surrounding childhood. Furthermore, uncertainty may be a key feature of family members’ experiences of chronic conditions, not just individuals with chronic conditions, as has been shown here. Lastly, it has been demonstrated that uncertainty is a broad concept and there are likely to be a number of uncertainties that contribute to the experience of chronic conditions.

## Notes

1 During absence seizures activity ceases; the person stares blankly and is unresponsive.

2 Tonic–clonic seizures begin with a tonic phase where the muscles contract so the person becomes stiff. The clonic phase then begins, which involves symmetrical jerking movements in the limbs as the muscles contract and relax.

3 Generalised seizures involve epileptic activity in both hemispheres of the brain. In contrast, focal seizures are located in one hemisphere of the brain.
